# Use of venovenous extracorporeal membrane oxygenation for perioperative management of acute respiratory distress syndrome caused by fat embolism syndrome: A case report and literature review

**DOI:** 10.1097/MD.0000000000024929

**Published:** 2021-02-26

**Authors:** Kenta Momii, Yuji Shono, Kanji Osaki, Yoshinori Nakanishi, Takeshi Iyonaga, Masaaki Nishihara, Tomohiko Akahoshi, Yasuharu Nakashima

**Affiliations:** a Emergency and Critical Care Center; b Department of Orthopaedic Surgery, Faculty of Medical Sciences, Kyushu University, 3-1-1 Maidashi, Higashi-ku; c Department of Orthopaedic Surgery, Nakabaru Hospital, 2-12-1 Befukita Shimemachi Kasuyagun, Fukuoka, Japan.

**Keywords:** acute respiratory distress syndrome, bilateral open femoral fractures, extracorporeal membrane oxygenation, fat embolism syndrome

## Abstract

**Introduction:**

Fat embolism syndrome (FES) is a known complication of long bone fracture and can affect multiple organs. The organ most commonly affected with FES is the lung. Severe cases of FES from long bone fracture can cause acute respiratory distress syndrome (ARDS). Although the treatment of ARDS remains challenging, it is reported that a lung protection strategy and prone positioning are effective. In addition, early fixation is reported to be beneficial in respiratory failure due to FES, though it may exacerbate respiratory failure during the perioperative period. We report the use of venovenous extracorporeal membrane oxygenation (VV-ECMO) for the successful perioperative management of a patient diagnosed with ARDS due to FES.

**Patient concerns:**

A 24-year-old man injured in a traffic accident was brought to our emergency department due to shock and consciousness disorder.

**Diagnosis:**

After examining the patient, we noted bilateral pneumothorax, liver and spleen injuries, and multiple long bone fractures. Four days after admission, he was diagnosed with FES due to a prolonged consciousness disorder, progressive hypoxia with diffuse lung damage, and cutaneous and mucosal petechiae.

**Intervention:**

As respiratory failure progressed, VV-ECMO was initiated on the 6th day. To improve the respiratory failure caused by ARDS, prone position therapy was necessary. Thus, we performed osteosynthesis on the 9th day under ECMO. Prone position therapy was started after surgery.

**Outcomes:**

Subsequently, his respiratory condition and chest radiographs improved steadily. VV-ECMO was discontinued on the 17th day and the ventilator was removed on the 28th day. His consciousness levels improved without residual central nervous system complications.

**Conclusion:**

Our study reveals the successful improvement of FES-induced ARDS by osteosynthesis and prone positioning under VV-ECMO. This strategy prioritizes supportive treatment over pharmacologic interventions.

## 1 Introduction

Fat embolism syndrome (FES) is a serious complication of long bone fracture and multiple trauma with a mortality rate of 5% to 20%.^[1]^ The treatment of fractures with FES is challenging. Severe FES can cause acute respiratory distress syndrome (ARDS) and requires multidisciplinary management.

Prone-position ventilation has significant benefits for patients with severe ARDS. It is commonly used to enhance oxygenation by improving pulmonary function.^[2]^ The use of lung-protective ventilation and prone positioning is reported to help patients with ARDS caused by FES.^[3]^ However, in FES cases with unstable pelvic fractures, comminuted vertebral fractures, or dislocated femoral fractures, it is necessary to achieve bone stability by performing early rigid internal fixation before placing the patient in the prone position. Numerous studies have shown that early definitive long bone fracture fixation is important in the prevention of respiratory complications.^[1,4]^

There is increasing evidence to support the use of venovenous extracorporeal membrane oxygenation (VV-ECMO) as a supportive treatment for severe ARDS. However, limited literature exists on the application of VV-ECMO for the treatment of ARDS secondary to FES and its use during osteosynthesis. We present an interesting case of ARDS, caused by FES due to bilateral femoral open fractures, which was successfully managed using VV-ECMO during osteosynthesis with postoperative prone-positioning.

## 2 Case presentation

Written informed consent was obtained from the patient for publication of this case report and any accompanying images.

**Figure 1:**
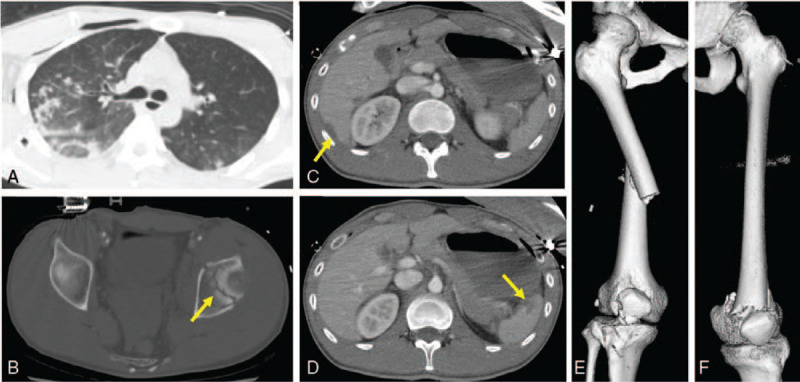
Computed tomography at the time of injury showing bilateral pneumothorax (A), left acetabular fracture (B), liver injury (C), spleen injury (D), right femoral fracture, patellar fracture, tibial plateau fracture (E), and left distal third femoral fracture (F).

**Figure 2:**
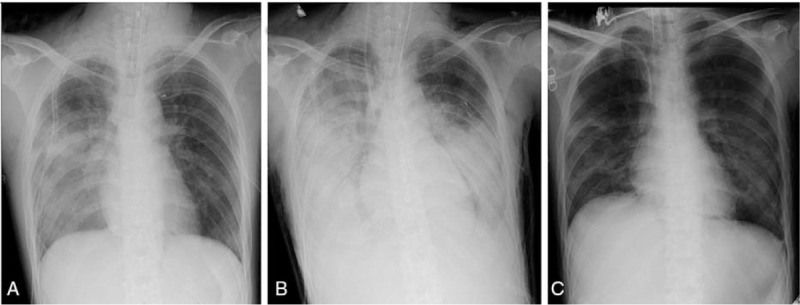
The chest radiograph on the 4th day after injury (A) showed progressive bilateral pulmonary infiltrate compared to the chest radiograph on the day of injury (B). On the 17th day after the injury (8th day after the osteosynthesis), the infiltrative shadow improved, and venovenous extracorporeal membrane oxygenation was withdrawn (C).

**Figure 3:**
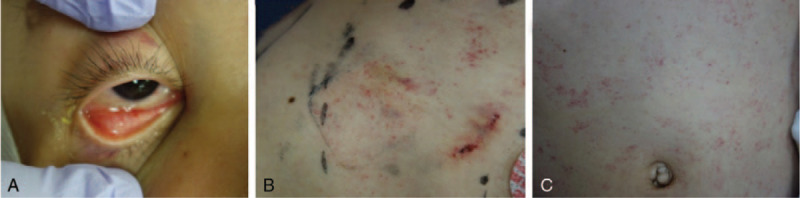
Multiple subconjunctival, precordial, and abdominal petechiae.

A 24-year-old man riding a motorcycle collided with a car while turning to the right. He fell off the motorcycle after the collision, and was injured by another car. He had no previous medical history. When he was brought to our emergency department in an ambulance, his airway was patent, he was tachypneic, hypotensive, tachycardic, and his Glasgow coma scale score was 7 (E1V2M4). Intubation, bilateral chest drainage, and blood transfusion were performed. Following assessment by whole-body computed tomography (CT), he was diagnosed with bilateral pneumothorax, liver and spleen injuries, fractures of the left acetabulum, right patella and tibial plateau, with open fractures of the shaft of the right femur and distal third of the left femur (Fig. 1). External fixation and irrigation of both thighs were performed in the operating room. The liver and spleen injuries were treated conservatively. After surgery, his consciousness disorder persisted. Initially, hypoxia was not accompanied by significant changes in chest radiographs; however, serial chest radiographs showed progressive bilateral pulmonary opacities consistent with ARDS (Fig. 2A and B). Contrast-enhanced CT also showed bilateral patchy gland glass opacity and consolidation matching chest radiographs, and there was no filling defect in pulmonary artery. Arterial blood gases on the 4th day of admission showed a PaO_2_/FiO_2_ (P/F) ratio of 100 mmHg under 10 cm H_2_O of positive end-expiratory pressure (PEEP). In addition, the patient developed subconjunctival, abdominal, and precordial petechiae (Fig. 3). He remained tachycardic, febrile with a body temperature >38°C, and was jaundiced. Laboratory data showed exacerbation of anemia (hemoglobin, 9.1 × 10 g/L), decreased platelet count (88 × 10^9^ /L), and elevated total bilirubin levels (8.9 × 10 μmol/ L). Gurd and Wilson criteria (3 major and 5 minor criteria) and Schonfeld scoring system were used for the diagnosis of FES. The criteria of both scoring systems were met, thereby supporting the positive diagnosis of FES (Table 1).^[5,6]^

**Table 1:** Diagnostic criteria of fat embolism syndrome.

Criteria	Findings	Points
Gurd criteria^∗^	Major	
	**Petechial rash**	
	**Respiratory insufficiency**	
	**Cerebral involvement**	
	Minor	
	**Tachycardia**	
	**Fever**	
	Retinal changes	
	Renal changes	
	**Jaundice**	
	**Anemia**	
	**Thrombocytopenia**	
	Elevated ESR	
	Fat macroglobulinemia	
		
Schonfeld criteria^†^	**Petechia**	5
	**Diffuse alveolar infiltrates**	4
	**Hypoxia (PaO**_**2**_ **<** **70 Torr)**	3
	**Confusion**	1
	**Fever (> 38**°C**)**	1
	**Tachycardia (> 120/min)**	1
	Tachypnea (> 30/min)	1

ESR = erythrocyte sedimentation rate.

∗ At least 1 major finding and 4 minor findings are needed for diagnosis.

† Cumulative score >5 required for diagnosis. The findings in bold are presented in our case.

**Figure 4:**
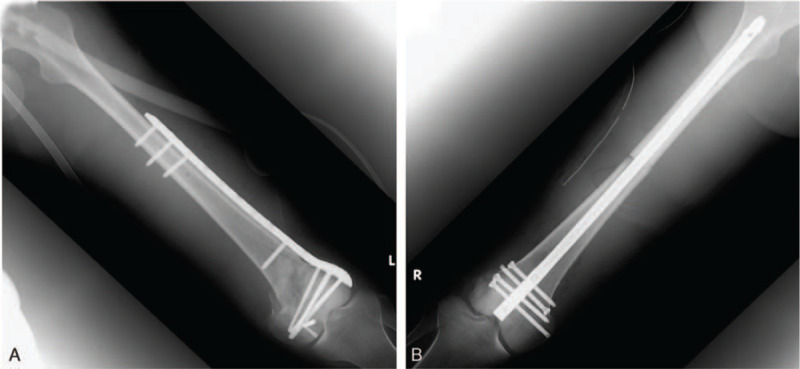
The left distal third femoral fracture was fixed with cannulated cancellous screws, headless screws, and a lateral locking plate (A). The right femoral fracture was fixed using a retrograde intramedullary nail (B).

The patient had type 1 respiratory failure which gradually worsened with conventional lung-protective ventilation. Due to a persistent consciousness disorder, prophylactic antiepileptic drug administration, anticerebral edema therapy, and targeted temperature management using an intravascular temperature control catheter were performed.^[7]^ On the 6th day of admission, the Murray score was 3.5 indicating severe lung injury: P/F ratio of 81 mmHg (4 points), pulmonary edema in all lung fields on chest X-ray (4 points), PEEP of 14 cm H_2_O (3 points), compliance of 23.3 ml/cm H_2_O (3 points). Because his cardiac function was not reduced and hemodynamic status was maintained, we chose VV-ECMO over venoarterial-ECMO.^[8]^ A 23 French multi-stage drainage cannula was inserted from the left femoral vein to the inferior vena cava, and a 17 French single-stage return cannula was cannulated from the right internal jugular vein to the superior vena cava. The ECMO rotation speed was 3300 r/minute and the flow rate was 4.5 L/minute with 100%FiO_2_ and 4.5 L/minute sweep gas flow. Under the condition, his peripheral oxygen saturation was maintained >95% and blood gas analysis showed a pH of 7.42, PO_2_ of 95 mmHg, and PCO_2_ of 46.6 mmHg. Although lung protection management was continued with high PEEP and low tidal volume after VV-ECMO introduction, improvement in respiratory condition was not observed. Therefore, we decided to convert the external fixation to internal fixation to begin prone position therapy. Osteosynthesis was performed on the 9th day of admission under VV-ECMO. In the supine position a tourniquet was applied on the left side. The lateral parapatellar approach was used to reduce the fracture and fix the screws and lateral locking plate (Fig. 4A). The tourniquet was released to place the screws in the proximal holes of the plate, following which there was continued congestive bleeding. Heparin had been introduced into the circuit at the beginning of the operation, but due to a large amount of blood loss, protamine sulfate, an antagonist for heparin, was administered midoperation. The bleeding was controlled by closing the left knee wound and inserting a drain. Osteosynthesis of the right femur was then performed using a retrograde intramedullary nail (Fig. 4B) without any serious complications. The total operation time was 4 hours and 29 minutes and the total blood loss amounted to 3800 g. Sixteen units of red blood cell transfusion, 20 units of platelet transfusion, and 16 units of fresh frozen plasma were needed. The ECMO circuit was run heparin free for 2 days postsurgery.

Prone-position ventilation was initiated on the second day postsurgery after the hemodynamics became stable. The patient was placed in the prone position for 16 hours a day. Low tidal volume ventilation (6 ml/kg) and an infusion of neuromuscular blockade was introduced. To address severe acute kidney injury due to hemolysis, we also started continuous renal replacement therapy on the 14th day. For 4 days, the patient was managed in the prone position. His respiratory condition and chest radiographs improved steadily over the 8 days following osteosynthesis, and VV-ECMO was discontinued on the 17th day (Fig. 2C). The ventilator was removed on the 28th day. At that time, his respiratory status improved to P/F ratio: 370, and multiple petechiae disappeared. His conscious level normalized without residual central nervous system complications.

## 3 Discussion

FES is often associated with orthopedic trauma. The pulmonary circulation is most commonly affected in FES, with up to 75% of patients experiencing respiratory depression.^[9]^ However, a definitive diagnosis of FES is not easy. While previously published large cohort studies have reported that the frequency of FES is 0.17% to 0.9% in patients with bone fracture, autopsy-based studies showed FES is present in 68% to 82% of blunt trauma patients.^[10–13]^ In this case, despite the prompt resuscitation of the chest injury, the patient decompensated into respiratory failure rapidly. In addition, the prolonged consciousness disorder, the subconjunctival and precordial petechiae, and the appearance of systemic inflammatory response syndrome contributed to the diagnosis of FES. Subconjunctival petechiae was the decisive factor in the diagnosis, as all other symptoms are nonspecific for patients with multiple trauma.

Fat embolism can be explained by 2 pathophysiologic mechanisms: the mechanical and biochemical theories.^[1,14]^ The mechanical theory suggests that obstruction of the pulmonary capillary beds occurs because of fat droplets released from the bone marrow into the venous system. The fat droplets travel through capillaries or arteriovenous shunts to the systemic vasculature. The mechanism is triggered by elevated intramedullary pressure following trauma. Occasionally, orthopedic procedures such as intramedullary nailing can increase the pressure and cause a second hit of FES. Alternatively, the biochemical theory proposes that trauma elevates inflammatory mediators and releases free fatty acids from the bone marrow into the venous system. Free fatty acids damage the endothelial cells of each organ and subsequently cause vasogenic edema and hemorrhage. This condition further promotes proinflammatory cytokine release and exacerbates inflammation.

Few pharmacologic interventions have proven to be effective for the treatment of FES. The use of hypertonic glucose to decrease free fatty acid mobilization, ethanol to decrease lipolysis, and dextran-40 to reduce the aggregation of platelets and erythrocytes do not show significant therapeutic effects in FES.^[15]^ In an experimental model of FES, heparin was demonstrated to increase pulmonary vascular permeability and reduce consumption coagulopathy.^[16]^ However, considering hemorrhagic complications, heparin is contraindicated in most patients with multiple trauma. Corticosteroids have been studied because their anti-inflammatory effects may suppress the biochemical theory-based organ and systemic damage. A recent meta-analysis suggests that corticosteroids are an efficacious prophylactic measure against FES.^[17]^ However, the use of corticosteroids may increase the risk of infection in open fractures. Therefore, we prioritized supportive treatment over pharmacologic interventions.

VV-ECMO has been used as a rescue therapy for severe respiratory failure for over 50 years. An observational study during the influenza A (H1N1) pandemic and a recently published meta-analysis support the application of VV-ECMO in the management of patients with severe ARDS.^[18,19]^ However, its efficacy remains controversial, and it is not easy to perform high quality randomized controlled trials due to the heterogeneity of cases. Additionally, VV-ECMO is only a supportive therapy and does not provide fundamental treatment. It permits the administration of ultra-protective ventilation strategies while awaiting an improvement in the patient's clinical condition.

Prone position therapy is a supplementary strategy for the treatment of ARDS with lung-protective ventilation. Compared to the supine position, the prone position provides more homogeneously distributed ventilation and perfusion. It results in recruitment of the dorsal side of the lung, increasing end-expiratory lung volume, decreasing alveolar shunting, and improving tidal volume.^[20]^ A large randomized controlled trial showed that prone position therapy reduces mortality in patients with severe ARDS.^[21]^ Furthermore, a recent meta-analysis suggested that prone position therapy was effective when carried out for at least 12 hours daily.^[2]^ In the present case, chest CT revealed massive dense consolidation in the dorsal side of the lungs bilaterally. Therefore, we performed rigid fixation to allow repositioning in the prone position and applied prone position therapy for 16 hours daily. Our patient showed rapid improvement in respiratory status after the start of prone position therapy and survived without any serious sequelae.

We performed a retrospective analysis of FES patients treated with ECMO reported in previous literature. The PubMed database was searched using the keywords “fat embolism syndrome” and “extracorporeal membrane oxygenation” Our search revealed 7 other reports on the subject (Table 2).^[22–28]^ Of the total 8 cases, 6 were trauma cases, including lower extremity fractures, and 2 were in lung transplantations from donors who were victims of trauma. VV-ECMO was given in 5 cases, and venoarterial extracorporeal membrane oxygenation (VA-ECMO) was performed in 3 cases. Respiratory and circulatory failures occurred preoperatively in 2 cases, intraoperatively in 2 cases, and postoperatively in 4 cases. Seven patients survived, and the duration of ECMO in survivors was 1 hour-17 days. Two cases developed obstructive shock intraoperatively suspected to be a result of the mechanical pathophysiology of FES, which improved promptly after VA-ECMO was initiated.^[26,27]^ The present case and 3 other cases developed respiratory failure consistent with ARDS.^[22,24,28]^ Since they improved gradually with the combination of VV-ECMO and a lung-protective strategy, ARDS was suspected to be a result of the biochemical pathophysiology of FES. The other 2 cases were donor-acquired FES after lung transplantation.^[23,25]^ One patient died early after transplantation due to primary graft dysfunction leading to multiorgan failure. Another patient recovered from ARDS due to FES and was weaned from VV-ECMO over 17 days. In these 2 cases, no image abnormalities suggestive of FES were noted in the donor's lungs before transplantation. Therefore, it was concluded that the biochemical pathophysiology of FES progressed rapidly after transplantation. Although the number of cases are limited, it can be inferred that even with fulminant FES, the outcome may not be poor if appropriate interventions are promptly undertaken.

**Table 2:** Case reports of fat embolism syndrome treated with extracorporeal membrane oxygenation.

	Age, Sex	Cause	Timing of FES onset	Modality of ECMO	Duration of ECMO	Outcome
Present case	24, M	Pelvic, bilateral femoral, and tibial fracture	4 days after injury	VV	11 days	Alive
Popovich et al, 2019^[22]^	24, M	Femoral, tibial, and fibular fracture	2 days after injury	VV	12 days	Alive
Jacob et al, 2016^[23]^	28, M	Lung transplantation from the donor of trauma	After operation	VV	17 days	Alive
Valchanov et al, 2014^[24]^	32, M	Traumatic lower limb amputation	Within 2 hours after operation	VV	6 days	Alive
López-Sánchez et al, 2010^[25]^	59, M	Lung transplantation from the donor of trauma	After operation	VA	25 hours	Died (45 hours after transplantation by MOF and massive bleeding)
Arai et al, 2007^[26]^	76, M	Femoral neck fracture	Intraoperation	VA	1 hour	Alive
Igarashi et al, 2006^[27]^	61, F	Femoral fracture	Intraoperation	VA	3 days	Alive
Webb et al, 2004^[28]^	38, M	Ulnar and femoral fracture	After operation	VV	5 days	Alive

ECMO = extracorporeal membrane oxygenation, F = female, FES = fat embolism syndrome, M = male, MOF = multiple organ failure, VA = venoarterial, VV = venovenous.

If there is severe ARDS due to FES, surgery cannot be performed safely because further embolism may occur during operative fixation. Therefore, we performed osteosynthesis under VV-ECMO. Popovich et al reported a case of FES due to floating knee injury treated with fracture fixation under VV-ECMO.^[22]^ The surgery was conducted successfully, with only 500 ml of blood loss without any circuit issues intraoperatively. One of the problems faced by our patient was intraoperative blood loss. Owing to the increased blood loss, the VV-ECMO circuit collapsed during surgery, requiring a large blood transfusion. The use of heparin and congestion associated with cannulation was the cause of this increased blood loss. Heparin was administered during the operation to prevent circuit blockage due to blood clots, however, it might be safer to stop heparin to prevent excessive intraoperative bleeding. Some reports suggest that stopping the heparin for a while is safe during ECMO.^[29,30]^ The patient was in a hypercoagulable state which needed exchange of ECMO before operation. In addition, the surgical field was on the same side as the ECMO cannulation, which is supposed to be difficult for safely exchanging the ECMO in the emergent situation. For these reasons, we performed the operation under heparization for ECMO in the present case. It is highly possible that contralateral cannulation reduced the amount of blood loss because the skin incision for nail entry would have been smaller and the operation time shorter.

## 4 Conclusions

We successfully treated our patient, who had FES-induced ARDS following bilateral femoral open fractures, using VV-ECMO during osteosynthesis and repositioning to a prone position. The control of intraoperative bleeding is an integral part of the management of these patients.

## Author contributions

KM, YS, KO, YN, TI, MN, and TA contributed to the treatment of the patient.

**Conceptualization:** Yuji Shono.

**Project administration:** Tomohiko Akahoshi, Yasuharu Nakashima.

**Supervision:** Kanji Osaki, Yoshinori Nakanishi, Takeshi Iyonaga, Masaaki Nishihara.

**Writing – original draft:** Kenta Momii.

**Writing – review & editing:** Kenta Momii.

## References

[1] Rothberg DL Makarewich CA. Fat embolism and fat embolism syndrome. J Am Acad Orthop Surg 2019;27:e346–55. 30958807 10.5435/JAAOS-D-17-00571

[2] Munshi L Del Sorbo L Adhikari NKJ. Prone position for acute respiratory distress syndrome. A systematic review and meta-analysis. Ann Am Thorac Soc 2017;14: Supplement_4: S280–8. 29068269 10.1513/AnnalsATS.201704-343OT

[3] Banerjee A Aggarwal R Soni KD. Prone positioning in a patient with fat embolism syndrome presenting as diffuse alveolar haemorrhage: new perspective. BMJ Case Rep 2020;13:e233452. 10.1136/bcr-2019-233452 PMC7066632 32161081

[4] Nahm NJ Vallier HA. Timing of definitive treatment of femoral shaft fractures in patients with multiple injuries: a systematic review of randomized and nonrandomized trials. J Trauma Acute Care Surg 2012;73:1046–63. 23117368 10.1097/TA.0b013e3182701ded

[5] Gurd AR. Fat embolism: an aid to diagnosis. J Bone Joint Surg Br 1970;52:732–7. 5487573

[6] Schonfeld SA Ploysongsang Y DiLisio R. Fat embolism prophylaxis with corticosteroids. A prospective study in high-risk patients. Ann Intern Med 1983;99:438–43. 6354030 10.7326/0003-4819-99-4-438

[7] Schmutzhard E Engelhardt K Beer R. Safety and efficacy of a novel intravascular cooling device to control body temperature in neurologic intensive care patients: a prospective pilot study. Crit Care Med 2002;30:2481–8. 12441758 10.1097/00003246-200211000-00013

[8] Murray JF Matthay MA Luce JM. An expanded definition of the adult respiratory distress syndrome. Am Rev Respir Dis 1988;138:720–3. 3202424 10.1164/ajrccm/138.3.720

[9] McCarthy B Mammen E Leblanc LP. Subclinical fat embolism: a prospective study of 50 patients with extremity fractures. J Trauma 1973;13:9–16. 4687250

[10] Bulger EM Smith DG Maier RV. Fat embolism syndrome. A 10-year review. Arch Surg 1997;132:435–9. 9108767 10.1001/archsurg.1997.01430280109019

[11] Eriksson EA Pellegrini DC Vanderkolk WE. Incidence of pulmonary fat embolism at autopsy: an undiagnosed epidemic. J Trauma 2011;71:312–5. 21825932 10.1097/TA.0b013e3182208280

[12] Mudd KL Hunt A Matherly RC. Analysis of pulmonary fat embolism in blunt force fatalities. J Trauma 2000;48:711–5. 10780606 10.1097/00005373-200004000-00020

[13] Stein PD Yaekoub AY Matta F. Fat embolism syndrome. Am J Med Sci 2008;336:472–7. 19092320 10.1097/MAJ.0b013e318172f5d2

[14] Husebye EE Lyberg T Roise O. Bone marrow fat in the circulation: clinical entities and pathophysiological mechanisms. Injury 2006;37: Suppl 4: S8–18. 16990064 10.1016/j.injury.2006.08.036

[15] Stoltenberg JJ Gustilo RB. The use of methylprednisolone and hypertonic glucose in the prophylaxis of fat embolism syndrome. Clin Orthop Relat Res 1979;143:211–21. 509829

[16] Burhop KE Selig WM Beeler DA. Effect of heparin on increased pulmonary microvascular permeability after bone marrow embolism in awake sheep. Am Rev Respir Dis 1987;136:134–41. 3300440 10.1164/ajrccm/136.1.134

[17] Bederman SS Bhandari M McKee MD. Do corticosteroids reduce the risk of fat embolism syndrome in patients with long-bone fractures? A meta-analysis. Can J Surg 2009;52:386–93. 19865573 PMC2769117

[18] Munshi L Walkey A Goligher E. Venovenous extracorporeal membrane oxygenation for acute respiratory distress syndrome: a systematic review and meta-analysis. Lancet Respir Med 2019;7:163–72. 30642776 10.1016/S2213-2600(18)30452-1

[19] Peek GJ Mugford M Tiruvoipati R. Efficacy and economic assessment of conventional ventilatory support versus extracorporeal membrane oxygenation for severe adult respiratory failure (CESAR): a multicentre randomized controlled trial. Lancet 2009;374:1351–63. 19762075 10.1016/S0140-6736(09)61069-2

[20] Gattinoni L Busana M Giosa L. Prone positioning in acute respiratory distress syndrome. Semin Respir Crit Care Med 2019;40:94–100. 31060091 10.1055/s-0039-1685180

[21] Guerin C Reignier J Richard JC. Prone positioning in severe acute respiratory distress syndrome. N Engl J Med 2013;368:2159–68. 23688302 10.1056/NEJMoa1214103

[22] Popovich I Singh V Vickery B. Perioperative support of a patient with fat embolism syndrome with extracorporeal membrane oxygenation. BMJ Case Rep 2019;12:e227747. 10.1136/bcr-2018-227747 PMC6536217 31092491

[23] Jacob S Courtwright A El-Chemaly S. Donor-acquired fat embolism syndrome after lung transplantation. Eur J Cardiothorac Surg 2016;49:1344–7. 26468269 10.1093/ejcts/ezv347 PMC6279222

[24] Valchanov KEA Fowles JA Parmar J. Veno-venous extracorporeal membrane oxygenation for fat embolism. J Med Cases 2014;5:488–90.

[25] López-Sánchez M Alvarez-Antoñán C Arce-Mateos FP. Single lung transplantation and fatal fat embolism acquired from the donor: management and literature review. Clin Transplant 2010;24:133–8. 19888997 10.1111/j.1399-0012.2009.01131.x

[26] Arai F Kita T Nakai T. Histopathologic features of fat embolism in fulminant fat embolism syndrome. Anesthesiology 2007;107:509–11. 17721255 10.1097/01.anes.0000278898.62036.5f

[27] Igarashi M Kita A Nishikawa K. Use of percutaneous cardiopulmonary support in catastrophic massive pulmonary fat embolism. Br J Anaesth 2006;96:213–5. 16377648 10.1093/bja/aei304

[28] Webb DP McKamie WA Pietsch JB. Resuscitation of fat embolism syndrome with extracorporeal membrane oxygenation. J Extra Corpor Technol 2004;36:368–70. 15679281

[29] Muellenbach RM Kredel M Kunze E. Prolonged heparin-free extracorporeal membrane oxygenation in multiple injured acute respiratory distress syndrome patients with traumatic brain injury. J Trauma Acute Care Surg 2012;72:1444–7. 22673280 10.1097/TA.0b013e31824d68e3

[30] Ried M Bein T Philipp A. Extracorporeal lung support in trauma patients with severe chest injury and acute lung failure: a 10-year institutional experience. Crit Care 2013;17:R110. 23786965 10.1186/cc12782 PMC4056791

